# Resolving the Electroencephalographic Correlates of Rapid Goal-Directed Chunking in the Frontal-Parietal Network

**DOI:** 10.3389/fnins.2019.00744

**Published:** 2019-07-19

**Authors:** Jiaoyan Pang, Xiaochen Tang, Qi-Yang Nie, Markus Conci, Peng Sun, Haibin Wang, Junlong Luo, Jijun Wang, Chunbo Li, Jing Luo

**Affiliations:** ^1^Shanghai Key Laboratory of Psychotic Disorders, Shanghai Mental Health Center, Shanghai Jiao Tong University School of Medicine, Shanghai, China; ^2^School of Government, Shanghai University of Political Science and Law, Shanghai, China; ^3^Department Psychologie, Ludwig-Maximilians-Universität München, Munich, Germany; ^4^Mental Health Education and Research Center, Shandong University of Finance and Economics, Jinan, China; ^5^School of Educational Science, Huangshan University, Huangshan, China; ^6^Department of Psychology, Shanghai Normal University, Shanghai, China; ^7^School of Psychology, Capital Normal University, Beijing, China; ^8^Key Laboratory of Mental Health, Institute of Psychology, Chinese Academy of Sciences, Beijing, China

**Keywords:** beta-gamma oscillations, goal-directed chunking, prefrontal-parietal network, parallel-factor analysis model, time-frequency analysis

## Abstract

Previous studies have revealed a specific role of the prefrontal-parietal network in rapid goal-directed chunking (RGDC), which dissociates prefrontal activity related to chunking from parietal working memory demands. However, it remains unknown how the prefrontal and parietal cortices collaborate to accomplish RGDC. To this end, a novel experimental design was used that presented Chinese characters in a chunking task, testing eighteen undergraduate students (9 females, mean age = 22.4 years) while recoding the electroencephalogram (EEG). In the experiment, radical-level chunking was accomplished in a timely stringent way (RT = 1485 ms, SD = 371 ms), whereas the stroke-level chunking was accomplished less coherently (RT = 3278 ms, SD = 1083 ms). By comparing the differences between radical-level chunking vs. stroke-level chunking, we were able to dissociate the chunking processes in the radical-level chunking condition within the analyzed time window (−200 to 1300 ms). The chunking processes resulted in an early increase of gamma band synchronization over parietal and occipital cortices, followed by enhanced power in the beta-gamma band (25–38 Hz) over frontal areas. We suggest that the posterior rhythmic activities in the gamma band may underlie the processes that are directly associated with perceptual manipulations of chunking, while the subsequent beta-gamma activation over frontal areas appears to reflect a post-evaluation process such as reinforcement of the selected rules over alternative solutions, which may be an important characteristic of goal-directed chunking.

## Introduction

Chunking, which refers to the integration of distinct pieces of information into a single unit, plays an important role in human memory, learning, and problem solving (Chase and Simon, 1973; Gobet et al., 2001). During spontaneous chunking like chess playing and motor sequence learning, the sequence of stimulus responses gradually translates into units with extensive practice (Tremblay et al., 2009, 2010). On the contrary, rapid goal-directed chunking (RGDC) can occur in a cognitively controlled way on the basis of existing knowledge (Miller, 1956). For example, one may realize that the seemingly meaningless letter string “fbiibm” actually consists of two familiar units, “FBI” and “IBM,” with these chunks in turn improving performance. Considering the different processes involved, it is important to know whether neural correlates underlying RGDC would be different from spontaneous chunking, which is critically mediated by the basal ganglia (Boyd et al., 2009; Jin et al., 2014).

Bor et al. (2003) and Bor and Owen (2006) have demonstrated that intentionally chunking separated dots into familiar objects particularly activated the prefrontal parietal network. In their spatial working memory task, participants had to maintain a dot trajectory within a 4 × 4 square space. Structured sequences, encouraging reorganization of these trajectories into coherent chunks like, for example letters, showed increased activation in prefrontal cortex and in the inferior parietal lobule during encoding, while unstructured trials (without chunking) showed increased activation in parietal and premotor cortices during the delay. This shows a successful dissociation of chunking from the maintenance of information in the frontal cortex, however, it remains unknown how the prefrontal cortex interacts with the parietal lobule during chunking, and the temporal characteristics of the cognitive processes underlying RGDC.

In our previous fMRI study on the chunk decomposition (Knoblich et al., 1999), the bilateral posterior parietal cortex (BA7/40) depicted a parametric activation that was related to an increasing tightness (tight > intermediate > loose). The tightness was classified based on the spatial relationship between to-be-moved parts and characters in an initial question phase, in loose condition, the two parts were spatially separated, and to-be-removed parts were peripherally adjacent to and embedded in the question characters in the intermediate and tight condition, respectively (Tang et al., 2016). Neuropsychological data has also shown that patients with parietal damage reveal impaired processes of object manipulation but not of object rehearsals in working memory (Koenigs et al., 2009). As to the visual cortex, local sensory information has been found to be processed by dorsal visual pathway in the parietal lobule during perceptual integration (Liu et al., 2017). For the mental reorganization, object manipulation and local sensory information processing functions mentioned above, it is reasonable to predict that the posterior parietal and occipital areas might be activated in order to bind distinct visual elements into unified chunks in RGDC.

In addition, notwithstanding the involvement of lateral prefrontal cortex in chunking processes, it is uncertain whether the selection, or, post-evaluation of a problem-solving strategy causes the activation in prefrontal cortex. For example, the dorsolateral prefrontal cortex is involved in rule selection, as demonstrated by beta-gamma oscillations (19–40 Hz) of the local field potentials (LFPs) (Buschman et al., 2012). On the other hand, the neuronal synchronization in the prefrontal cortex possibly reflects the post-evaluation of a given rule, especially in a novel situation. For instance, some studies show that beta-gamma power plays an important role in reinforcement learning and in reward processing (Engel and Fries, 2010; Wang, 2010; Marco-Pallarés et al., 2015). To decide between these alternatives, the current study investigated the temporal relationship between oscillations in frontal and parietal cortices.

**FIGURE 1:**
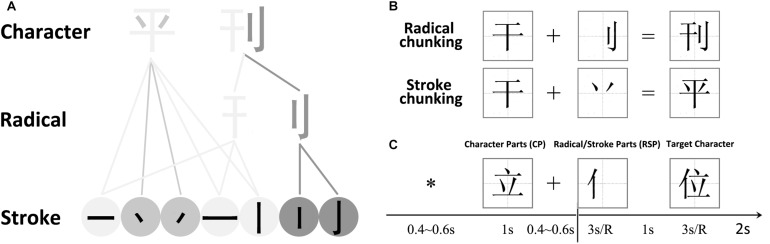
**(A)** Illustration of how a Chinese character is formed. A single stroke is the most basic and simple unit of radicals and characters. A radical is a sub-chunk of a character with some implied meaning or pronunciation. **(B)** Chunking Types. There were 2 chunking conditions, which were classified according to the parts that were to be combined – radicals or strokes. **(C)** Trial sequence. Subjects were asked to combine the Character Parts (CP, such as “
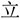
”) with the Radical/Stroke Parts (RSP, such as “
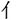
”) to form a new character (“
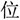
”). Each trial started with a central asterisk, followed by the Character Part and a fixation. The Radical/Stroke Parts appeared on the screen for at most 3000 ms until a response key was pressed, followed by an answer character.

The major aim of the present EEG study is to resolve the specific role of the prefrontal-parietal network during RGDC. We adopted a novel Chinese-character task with radical-level (regular) and stroke-level chunking (irregular, Figure 1). The contrast of regular versus irregular chunking could be especially helpful for EEG to resolve the brain’s spatiotemporal information processing capacity during RGDC, which would also control for confounding factors like task demand or difficulty, and even meaningful/meaningless features of the mental representation.

On the basis of the above theoretical reflections on chunking and the characteristics as provided by spectral analysis techniques, we predict that the process of binding elements into a chunk would initiate an increase of induced gamma band activity in the parietal lobe (Gray et al., 1989; Singer and Gray, 1995; Singer et al., 1997). Moreover, we assume that the post-evaluation of the problem-solving strategy would reveal beta-gamma band activity in frontal cortex at a later stage of problem-solving.

## Materials and Methods

### Participants

Eighteen undergraduate students (9 females, mean age = 22.4 years) were recruited from China Agricultural University and Beijing Forestry University and were paid for their participation. All were native Chinese speakers who had normal or corrected-to-normal vision and were right-handed. None of the participants were familiar with the five-stroke input method in Chinese. In the five-stroke input method, Chinese characters are encoded according to the stroke and font style, when using this input method, people have to decompose each Chinese character into strokes firstly, so participants familiar with the five-stroke input method have lots of experience in chunk decomposition. The decomposition of characters used in the five-stroke input method may interfere with our experimental effects. Written informed consent was obtained from each participant and the research protocol were approved by the Ethics Committees of the Institute of Psychology, Chinese Academy of Sciences in accordance with the with the Declaration of Helsinki.

### Materials

Chinese characters containing radicals and strokes constitute of a spatial rather than a phonological system, and this orthographic structure thus makes Chinese characters ideal examples of perceptual chunks (Tan et al., 2005; Luo et al., 2006). We therefore used a Chinese character-chunking task to investigate the cognitive processes of regular and irregular chunking (Figure 1A). The *stroke* represents the most fundamental elements of a character and carries no meaning. Therefore, the *stroke* itself cannot be treated as a separate unit/chunk. The *radical* in turn conveys certain semantic meanings or pronunciations of the character, and can thus serve as the most basic unit/chunk of a Chinese character. These structures inherent in Chinese characters allowed us to define two kinds of chunking or variants of “de-chunking” (Ohlsson, 1984; Ohlsson, 1992; Knoblich et al., 1999) manipulations, in order to investigate the neural correlates of regular and irregular chunking (Luo et al., 2006; Wu et al., 2009, 2010, 2013; Huang et al., 2015, 2018; Zhang et al., 2015; Tang et al., 2016). For the *radical-level chunking*, a *radical* needs to be jointed into the target to form a new Chinese chunk in a step-by-step manner, thereby revealing a regular chunking process. In case of *stroke-level chunking*, a stroke needs to be jointed into the target in an unusual way in order to result in a new Chinese chunk, such that *stroke-level chunking* may be considered a form of irregular chunking. The example in Figure 1 illustrates this difference of the chunking variants: Suppose we have the target word “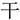,” it is easy to join the radical “
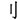
” into the target and get a new chunk “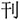,” because“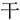,” and“
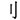
” are all meaningful chunks, and “
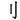
” doesn’t inserted into “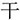,” they constitute a loose chunk. This is one example of the *radical-level* regular chunking. However, when we have the same target word “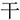,” it will be difficult to join a “
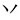
” to the “
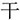
” to form the new chunk“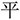,” because the strokes “
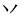
” themselves are not meaningful units and the way to insert them into “
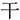
” to form “
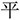
” is not straightforward. This is an example of the *stroke-level* irregular chunking. In sum, in this study, we studied the cognitive brain processing procedure involved in RGDC by contrasting the stroke-level irregular chunking with radical-level regular chunking. This design allows us to evaluate prefrontal, parietal involvement in RGDC in the “easy task > difficult task” contrast while controlling for the confounding factor of task demand or task difficulty as evident in a previous study (Bor et al., 2003). At the same time, other possible confounding factors such as the meaningful/meaningless, namable/unnamable, and successful/unsuccessful features of the mental representation can also be controlled.

A total of 350 characters were selected from the most commonly used 4754 characters in the Modern Chinese Frequency Dictionary (Cidian, 1986). The stimuli were real characters (CP, Character Part) and radicals or strokes (RSP, Radical, or Stroke Part). To eliminate any prediction of chunking type as potentially derived from the, we avoided using combined characters as CPs. Depending on the type of RSPs, the two chunking conditions in the present experiment are referred to as stroke chunking and radical chunking, respectively (Figure 1B). There were 70 trials in each of the two chunking conditions with overall 140 CPs, 140 RSPs, and 140 resulting target characters. An additional set of 70 trials replaced the RSP with a circle to issue a “No” response in order to counteract a potential automatic response inclination. The CPs and target characters were presented in black at the center of a white square subtending 75 × 75 pixels on a monitor screen. The RSPs appeared in the square at the location where they would subsequently appear in the target characters. There were 210 trials in total, all possible conditions presented in random order across 5 blocks of 42 trials each.

### Experimental Design

Participants were seated in a sound-attenuated and electrically shielded booth. Stimuli were delivered on a monitor at a distance of approximately 60 cm. Participants were instructed to decide whether a CP could be combined with an RSP, or circle to form another new character. CPs and RSPs were successively presented at the center of the screen. After an initial instruction message, participants were given 20 practice trials to ensure familiarity with the task and the button assignments. During the experiment, participants were asked to try to think efficiently in their mind instead of handwriting, and then to respond as fast and accurately as possible. Participants were informed to keep their head still during the formal experiment, with their eyes fixating the screen center.

On each trial (Figure 1C), a fixation asterisk was presented on the computer screen randomly from 400 to 600 ms to indicate the start of a new trial. The star was then replaced by a CP for 1000 ms, followed by a fixation cross, which was presented for a random interval between 400 and 600 ms. The RSP then appeared on the screen and remained visible for 3000 ms or until a button was pressed. During this period, participants were instructed to combine the RSP with the CP to generate a new, valid Chinese character in their mind. If participants were able to join the Character, they were instructed to tap a button “1” with their right index finger immediately after its generation, otherwise, when no solution was found, they were instructed to press the button “2” with their right middle finger. After the presentation of the RSP, the correct answer for each trial was presented for 3000 ms, which was followed by a blank screen for 2000 ms. Participants were asked to compare their own answer with the correct answer and to press the “1” or “2” keys on the keyboard to indicate if their solution agreed or disagreed with the provided answer, respectively. When they failed to solve the problem in the time given, participants were instructed to withhold their response. E-prime 2.0 (Psychology Software Tools, Inc.) was used to present the stimulus sequence and to collect the responses.

### Electroencephalogram (EEG) Recording and Analysis

The electroencephalogram (EEG) was collected from a 64-channel QuickCap (Compumedics United States of America, Charlotte, NC, United States) system with a Neuroscan SynAmps amplifier, transformed to averaged mastoids references (M1 and M2) during off-line processing. The vertical electrooculogram (VEOG) was recorded using electrodes placed below and above the left eye and the horizontal electrooculogram (HEOG) was recorded with electrodes placed left and right of the two eyes. EEG recordings were amplified through a 0.05–100 Hz bandpass filter and were digitized at a sampling rate of 500 Hz. Impedances of all electrodes were kept below 5 kΩ. The removal of ocular artifacts was accomplished by a regression procedure implemented in the Neuroscan software (Semlitsch et al., 1986). Continuous EEG recordings were segmented into epochs of 2000 ms subsequent to RSP onset plus a 500 ms pre-stimulus baseline. Epochs with voltage variations larger than 75 μV and error trials were excluded from the final analysis.

To estimate the induced activity, preprocessed epochs were wavelet-transformed using a complex Morlet wavelet implemented in the ERPWAVELAB toolbox (Mørup et al., 2007) with a center frequency of 1 and bandwidth parameter set to 1 (corresponding to a width of 6.28 cycles). The center frequency ranged from 1 to 70 Hz with steps in 1 Hz intervals. The analysis time window started from −500 ms before RSP onset until 2000 ms with 8 ms between each time point. To avoid edge artifacts, the windows of interest was then cut into epochs of −200 to 1300 ms, since lateralized readiness potentials (LRPs) can be considered to precede the manual reaction times (RTs, 1485 ms) by about 200 ms (Rinkenauer et al., 2004). The induced activity was normalized through dividing the post-stimulus power by the mean amplitude during baseline (−200 to 0 ms).

### Statistical Analysis

An exploratory analysis was performed to detect differences related to chunking. To this end, an ANOVA of difference was decomposed using the parallel-factor analysis (PARAFAC) model (Mørup et al., 2006; Montes et al., 2008; Verleger et al., 2013; Yokota et al., 2016), which yielded components across channel, frequency and time dimensions. The ANOVA was conducted to compare differences between the chunking conditions at the stage of RSP presentation. The resulting *F*-test value array was subsequently decomposed with the PARAFAC method to obtain components that show the most robust difference between conditions. The reason to use such an exploratory analysis was that the maximum differences between conditions are more difficult to extract from the plots of multi-channel time-frequency data than a specific electrode plot of time-frequency data. The number of components was set to 2 with iterations of 10000 and a sparsity of 0.1. Moreover, it should be noted that it was not possible to separate the localized maxima by increasing the component number to 3 or 4, in which case the first component would still reveal two topographical maxima. The components obtained from the PARAFAC model applied on *F* values are two-dimensional arrays of loadings corresponding to time and frequency in addition to spatial maps. The larger loading values in the two-dimensional arrays indicate larger differences between conditions at the given time and frequency, while the spatial maps reflect the scalp topography of these differences. According to the signature, the secondary quantification was conducted in the chosen frequency range, time window and channels just as manual inspection of the grand average in ERP analysis.

Based on the components of the PARAFAC analysis, a three-way repeated-measures ANOVA with the factors chunking type (Radical vs. Stroke), hemisphere (right vs. left) and electrodes was conducted on the average of the corresponding induced activity. On the basis of the topographic map of component 1, 20 electrodes over frontal cortex (left hemisphere: F1, F3, F5, F7, FC1, FC3, FC5, FC7, AF3, AF7, and right hemisphere: F2, F4, F6, F8, FC2, FC4, FC6, FC8, AF4, AF8) were selected for statistical analysis of the induced activities in the beta-gamma band around 27–37 Hz at 400–496 ms. For component 2, the statistical analysis was conducted on the average gamma activities of 48–58 Hz at 346–422 ms over occipital areas (left hemisphere: CP1, P1, P3, P5, P7, PO3, PO5, PO7, O1, and right hemisphere: CP2, P2, P4, P6, P8, PO4, PO6, PO8, O2). In order to avoid the issue of double dipping, the selected windows of interest for the time and frequency domain was larger than that of the maximum difference shown by the PARAFAC map, which would reduce the possibility of false positives. Furthermore, an ANOVA-type statistic for repeated-measures designs was implemented with wild bootstrap resampling for 1000 times using the R package MANOVA.RM, which improves the general applicability in a repeated-measures design with a relatively small sample (Friedrich et al., 2017).

To further understand the functional significance of EEG power effects, we estimated source activity of chunking after the onsets of RSP parts in the beta-gamma and higher gamma frequency band using the dynamic imaging of coherent sources (DICS) beamforming approach (Gross et al., 2001). By using DICS, a spatial filter was created based on the cross-spectral density matrices, which were computed separately for the beta-gamma and higher gamma oscillations. Using a multitaper fast Fourier transform (FFT), the power at the center frequency of 32 Hz (beta-gamma: ±10 Hz smoothing with 1 Slepian tapers) and 53 Hz (gamma: ±10 Hz smoothing with 1 Slepian tapers) was computed in separate time intervals between 400–496 ms and 346–422 ms. A common spatial filter was also constructed from the combined conditions. Using a realistically shaped 3-layer boundary elements volume conduction model, the lead field was calculated for each grid point with a 1-cm resolution derived from a standard MRI template brain provided by FieldTrip. Then, projections of power changes at the source level were obtained by applying the common filter separately for the two conditions. Statistical results of vertex-wise *t*-tests were cluster-based and were displayed on the standard Montreal Neurological Institute (MNI) brain.

## Results

### Behavioral Results

The accuracy rate of the radical and stroke chunking conditions was 99% (SD = 1%) and 84% (SD = 11%), respectively, revealing a statistically significant difference [*t*(17) = −6.00, *p* < 0.001, paired *t*-test]. This demonstrates that participants were more accurate in combining radicals than strokes with the respective character parts. The reaction times (RTs) for all correct trials in the radical and stroke chunking conditions were 1485 ms (SD = 371) and 3278 ms (SD = 1083), respectively. This difference in RTs was again significant [*t*(17) = −9.29, *p* < 0.001, paired *t*-test], and shows that participants needed significantly more time to successfully combine strokes with character parts to form a new character than in the case of radicals. The consistent difference in both accuracy and RT measures additionally also rules out any potential speed-accuracy tradeoffs. Within the time window of interest (from RSP display onset until 1300 ms), we assume that the main chunking processes in the radical chunking condition may have been accomplished given the relatively short mean RT (1485 ms). On the other hand, chunking in the stroke chunking condition (RTs = 3278 ms) would probably not have been completed in this time window. Since we are interested in the neural correlates of chunking, the radical chunking condition is ideal for the current analyses due to its consistency in accuracy (SD = 0.01) and in RTs (SD = 371), while, the stroke chunking condition serves as an ideal control with no (or at least, less) chunking occurring within the to-be-analyzed time window. Therefore, the relative increase of oscillatory power may be linked to chunking processes without an influence by response-related activity.

### EEG Results

**FIGURE 2:**
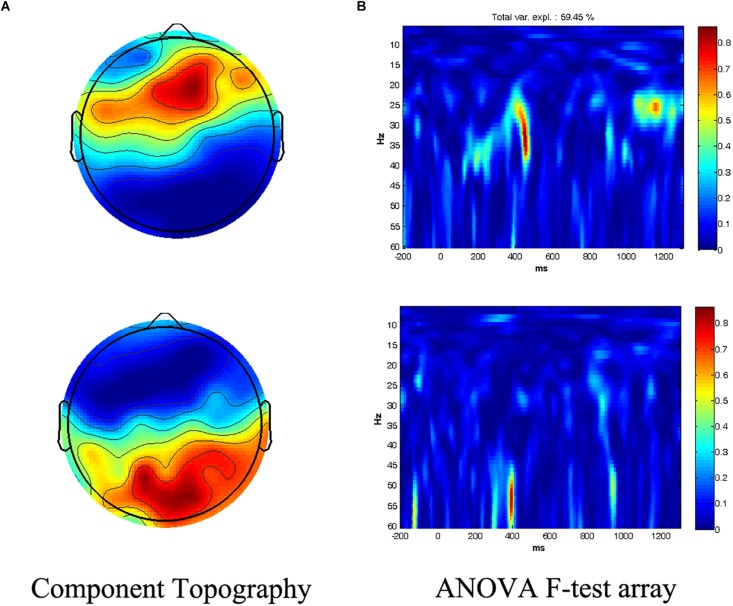
Differences between the radical and stroke chunking condition, as obtained through the parallel-factor analysis (PARAFAC) model. **(A)** Topography of the resulting 2 components, which show the largest difference during the chunking processes with frontal and occipito-parietal peaks, respectively. **(B)** Spectral and temporal characteristics of each component are displayed with their relative intensity.

The ANOVA *F*-test array was decomposed by PARAFAC to 2 components, which covered 59.45% of the variance between radical vs. stroke chunking conditions. Component 1 peaked in the frequency range at 33 Hz at a latency of 452 ms over electrode F2, while component 2 revealed a difference in the gamma band at around 53 Hz at a latency of 396 ms over electrode POZ (Figure 2). These maximum values of each component were then used to determine the windows of interest (WOI) for further analyses.

**FIGURE 3:**
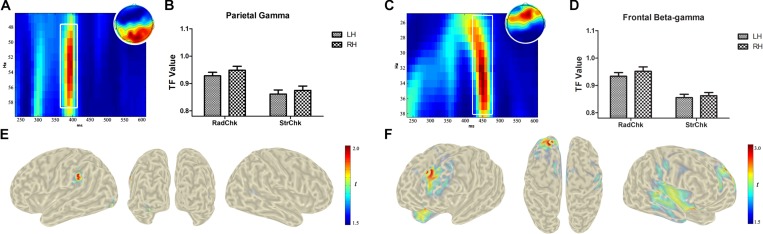
Electroencephalogram (EEG) power difference and source localization of the chunking effect. According to the signature of the differences, the time-frequency map was zoomed to highlight the analyzed time window (white boxes) for gamma activities of 48–58 Hz at 346–422 ms over occipital electrodes **(A)** and beta-gamma band around 27–37 Hz at 400–496 ms **(C)**. The bar plot of induced activities was calculated from the raw time-frequency values corresponding to frequency range and time window and displays respective activities for the gamma **(B)** and beta-gamma **(D)** band. Source activity of chunking after the onsets of RSP parts in the higher gamma and beta-gamma frequency band are localized in the left inferior parietal lobule (BA40, MNI: –58, –42, 33) and left inferior occipital gyrus (BA18, MNI: –34, –91, –10); **(E)** in left superior frontal gyrus (BA9, MNI: –22, 41, 36) and **(F)** in the orbital part of the right inferior frontal gyrus (BA47, MNI: 21, 16, –22).

As hypothesized in the Introduction, there was an increase in spectral power in the gamma band during the process of chunking [Radical > Stoke, *F* (1,17) = 11.25, *p* = 0.004, repeated-measures ANOVA, and *p*-WildBS < 0.001, wild bootstrap resampling of repeated-measures ANOVA, see Figures 3A,B], and there was no significant difference in the main effect of hemisphere or electrode and its corresponding interactions. The gamma power increase in the Radical chunking condition was driven by sources in the left inferior parietal lobule (BA40, MNI: −58, −42, 33) and left inferior occipital gyrus (BA18, MNI: −34, −91, −10) (Figure 3E). With regard to the average induced beta-gamma responses (Figures 3C,D), the statistical result also revealed a significant main effect of chunking type [Radical > Stoke, *F*(1,17) = 23.57, *p* < 0.001, and *p*-WildBS < 0.001], a marginally significant main effect of Electrode [*F*(6,102) = 2.47, *p* = 0.028 and *p*-WildBS = 0.055], and a three-way interaction effect [*F*(6,102) = 3.61, *p* = 0.003, and *p*-WildBS = 0.038]. Source localization indicated a prominent increase of beta-gamma oscillatory power in left superior frontal gyrus (BA9, MNI: −22, 41, 36) and in the orbital part of the right inferior frontal gyrus (BA47, MNI: 21, 16, −22) (Figure 3F).

## Discussion

Our EEG study sheds light on the neuronal time-frequency pattern of goal-directed chunking in the gamma and beta-gamma band in the prefrontal parietal network. To this end, we used a paradigm that required participants to combine a Chinese character with additional parts (radicals or strokes), and which revealed that the RGDC process evoked greater brain oscillations than the more demanding processes. This finding mirrors a previous study, which employed a strategic chunking manipulation in a random digit span working memory task (Bor and Owen, 2006). In agreement with fMRI findings of consistent co-activation of the prefrontal and parietal cortices during chunking (Bor et al., 2003; Bor and Owen, 2006; Bor and Seth, 2012), the observed increase of power in the gamma (48–58 Hz) frequency range was localized in the left inferior parietal lobule and in the left inferior occipital gyrus. Source locations of the subsequent beta-gamma band (25–38 Hz) enhancement in turn revealed an involvement of the left superior frontal gyrus and the orbital part of the right inferior frontal gyrus.

The gamma band oscillations observed between 270 and 410 ms over posterior occipital and parietal areas may directly link to the chunking processes that integrated the character parts and radicals into a coherent character representation. This might indicate that the parietal lobules contribute to the chunking processes by determining the relations of elements, such as their spatial relationship. The parietal lobe has indeed been found to reflect the mental manipulation of information in working memory, such as during perceptual binding (Rodriguez et al., 1999; Trujillo et al., 2005; Honkanen et al., 2014; Chen et al., 2018). For example, the perceptual completion of Mooney faces, which shares many similarities with our chunking task, requires to combine face pieces into to-be-identified wholes and this integration of parts into a coherent whole face revealed enhanced gamma band amplitudes in parietal cortices (Moratti et al., 2014).

Furthermore, the gamma band activity over occipital cortex is hypothesized to reflect the representation of a Chinese character, which may indicate the completion of perceptual processing by re-activation of visual cortices. According to the match-and-utilization (MUM) model, repeated perception of a stimulus would result in strengthened synaptic connections between neurons and stimuli that match with memory representations, thus leading to a significant activation of neuronal assemblies (Herrmann et al., 2004). In support of this model, meaningful words have consistently been found to evoke higher gamma band activity than non-words (Lutzenberger et al., 1994; Krause et al., 1998; Pulvermüller et al., 1999). In addition, the MUM model has particularly stressed that induced gamma band responses could also reflect memory matches when certain factors delay the processing of visual details, which is consistent with the chunking process as observed in the present study. Therefore, synchronization of gamma band activity at about 400 ms accords with findings that the activation of low-level visual cortex occurs at a relatively late stage of coherent percept formation (Jiang et al., 2008; Moratti et al., 2014).

In sum, we hypothesize that, during chunking, the target characters are represented by gamma oscillations over occipital areas as maintained via feedback projections from the parietal lobe. Induced gamma rhythm has been suggested as a putative co-activation mechanism for anatomically distributed neuronal assemblies during perceptual integration process (Keil et al., 1999; Tallon and Bertrand, 1999; Kaiser and Lutzenberger, 2003). During the course of chunking, the rhythm of gamma may thus help to establish the basis of communication between visual and parietal cortices. For instances, Mooney Face stimuli perception reveals a top-down modulation of gamma band oscillations in early visual cortex by feedback coupling with the parietal lobule (Liu et al., 2017), and additional feedforward connectivity from visual to parietal cortices (Moratti et al., 2014).

While the posterior rhythmic activities in the gamma band may underlie the processes that are directly associated with a perceptual manipulation of chunking, the immediately subsequent beta-gamma (25–38 Hz) activity over frontal area appears to reflect the positive reinforcement of the selected strategy, or of corresponding rules during the RGDC process. The observed lag of activation of the frontal lobes relative to parietal and visual cortices excludes an alternative explanation that the frontal lobes are responsible for monitoring the processes of chunking and/or are engaged in the selection of problem solving strategies. Rather, when considering the temporal relationship, beta-gamma band activity most likely resulted from a post-evaluation of the current performance. In addition, similar neural correlates may underlie the successful memory formation and retrieval in various tasks (Xue et al., 2010; O’Bryan et al., 2018). It has been demonstrated that different neuronal ensembles in the dorsolateral prefrontal cortex of monkeys responded to color- and orientation-based rules, as shown by a concurrent increase in beta-gamma synchronization (19–40 Hz) of the local field potentials (Buschman et al., 2012). The similarity of our EEG results with these findings in terms of frequency range and spatial location thus supports the assumption of beta-gamma band oscillations as paying a major role in rule reinforcement (Li et al., 2016). Moreover, according to a model proposed by Marco-Pallarés et al. (2015), synchronizations in the beta-gamma band serve to reinforce the current response set through an interaction with hippocampal and striatal regions, as supported by a combined EEG and fMRI study using a joint Independent Component Analysis (ICA) approach (Mas-Herrero et al., 2015).

The findings reported here may also demonstrate a distinction between goal-oriented and automatic chunking. On the one hand, the parietal involvement indicates the need to consciously manipulate the pieces in goal-oriented chunking in order to determine their spatial relationship, which differs from automatic perceptual chunking like word or character recognition (Pak et al., 2005). On the other hand, the activation of beta-gamma band activity may link to a post-evaluation process in goal-directed chunking such as rule reinforcement. Beta-gamma activity has previously been related to reward signals in decision-making tasks (Cunillera et al., 2012; Marco-Pallarés et al., 2015; Li et al., 2016), in that the monetary reward, depended on the temporal preciseness of the button press, causing an increase of power between 20 and 30 Hz. However, such an increase of beta-gamma power disappeared when the reward was randomly delivered such that no rule could be extracted (Keil et al., 2001). Similarly, when a new rule had to be inferred by trial and error in a modified version of the Wisconsin Card Sorting Task, then only the first positive feedback that indicated the correctness of the selected rule elicited an increase in the beta-gamma activity (Cunillera et al., 2012). In addition, Bor et al. (2003) demonstrated that automatic chunking like recognition of familiar shapes fails to induce the lateral prefrontal activity, which was prominent in goal-directed chunking, and they suggest greater activity in lateral PFC is related to facilitation of memory encoding.

## Conclusion

Our results provide insight into the unique characteristics of goal-directed chunking. Although it takes a shorter amount of time to combine a Chinese character with a radical (than with a stroke), a significant increase of frontal beta-gamma oscillations was observed subsequent to the initial gamma responses over parietal and occipital areas. In our framework of chunking, the induced gamma oscillations over occipital areas reflected the internal representation of the Chinese character, while parietal lobules help to determine the spatial relationship of elements during chunking. The subsequent frontal beta-gamma (25–38 Hz) activity were in turn interpreted as a reinforcement signal to promote the selected strategy or rule in a given problem solving situation.

## Ethics Statement

This study was carried out in accordance with the recommendations of Ethical Conduct for Research Involving Humans (the TCPS-2), Research Ethics Boards at the Ethics Committees of the Institute of Psychology, Chinese Academy of Sciences with written informed consent from all subjects. All subjects gave written informed consent in accordance with the Declaration of Helsinki. The protocol was approved by the Ethics Committees of the Institute of Psychology, Chinese Academy of Sciences.

## Author Contributions

JP, XT, and JL contributed to the conception and design of the study. JP, XT, and Q-YN collected the data. XT, PS, HW, and JuL performed the statistical analysis. JP and XT wrote the first draft of the manuscript. MC, CL, JW, and JL wrote sections of the manuscript. All authors contributed to manuscript revision, read and approved the submitted version.

## Conflict of Interest Statement

The authors declare that the research was conducted in the absence of any commercial or financial relationships that could be construed as a potential conflict of interest.

## Abbreviations

CP: Character Part
RGDC: rapid goal-directed chunking
RSP: Radical or Stroke Part.
